# Identification of somatic mutations in single cell DNA-seq using a spatial model of allelic imbalance

**DOI:** 10.1038/s41467-019-11857-8

**Published:** 2019-08-29

**Authors:** Lovelace J. Luquette, Craig L. Bohrson, Max A. Sherman, Peter J. Park

**Affiliations:** 1 Department of Biomedical Informatics, Harvard Medical School, Boston, MA USA; 2 Ludwig Center at Harvard, Boston, MA USA

**Keywords:** Genomics, Statistical methods

## Abstract

Recent advances in single cell technology have enabled dissection of cellular heterogeneity in great detail. However, analysis of single cell DNA sequencing data remains challenging due to bias and artifacts that arise during DNA extraction and whole-genome amplification, including allelic imbalance and dropout. Here, we present a framework for statistical estimation of allele-specific amplification imbalance at any given position in single cell whole-genome sequencing data by utilizing the allele frequencies of heterozygous single nucleotide polymorphisms in the neighborhood. The resulting allelic imbalance profile is critical for determining whether the variant allele fraction of an observed mutation is consistent with the expected fraction for a true variant. This method, implemented in SCAN-SNV (Single Cell ANalysis of SNVs), substantially improves the identification of somatic variants in single cells. Our allele balance framework is broadly applicable to genotype analysis of any variant type in any data that might exhibit allelic imbalance.

## Abstract (web summary)

Single cell whole-genome sequencing data harbors information about somatic genetic variation but is challenging to analyze. Here, the authors develop a spatial model to correct for allelic amplification imbalance and a somatic SNV genotyper SCAN-SNV for analyzing single cell DNA sequencing data.

## Introduction

Single-cell DNA sequencing (scDNA-seq) has recently emerged as an efficient and scalable tool to study genetic heterogeneity in multicellular organisms. Although whole-genome sequencing of bulk tissues has been used to identify somatic mutations, its sensitivity for mutations present in a low fraction of cells is limited. For example, mutations shared by fewer than 5% of cells are difficult to detect even with 100 × sequencing. scDNA-seq, on the other hand, offers the possibility of detecting mutations of essentially any frequency as long as they are present in the selected single cell. In addition, co-occurrence patterns among multiple mutations across multiple single cells can define subclonal populations and reveal evolutionary dynamics within a cell population. Recent applications of scDNA-seq have elucidated subclonal evolution processes in breast carcinoma^1^ and revealed a biological mechanism capable of generating chromothripsis, disastrous DNA damage often observed in cancer cells^2^. In our own work, we have used scDNA-seq to reconstruct cellular lineage in the brain using somatic mutations as markers^3^ and to uncover the accumulation of single nucleotide mutations in aging human neurons^4^.

**Fig. 1:**
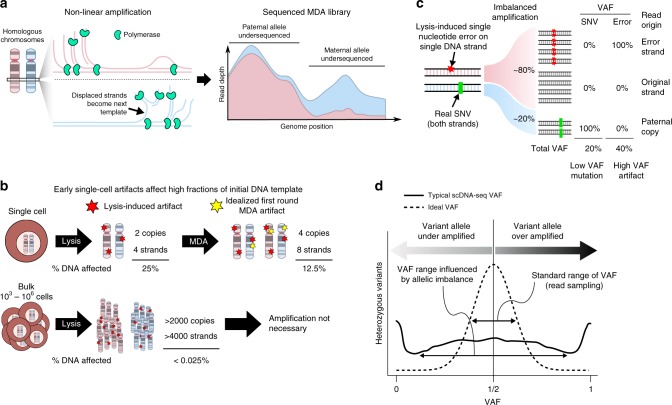
scDNA-seq artifacts. scDNA-seq analysis is complicated by a combination of imbalanced amplification of homologous alleles (allelic imbalance) and early artifacts that affect a large fraction of initial DNA. **a** MDA is a non-linear amplification process. DNA strands displaced by replication are immediately available for repeated rounds of replication, which can lead to imbalanced amplification between homologous alleles. Single cell sequencing depth is the sum of allele-specific sequencing depths, represented by a stacked depth plot. Pink: sequencing depth of maternal allele; blue: paternal allele. **b** Routine DNA damage during extraction protocols can disproportionately impact single cell DNA. Bulk DNA damage is mostly washed out since spontaneous errors are unlikely to recur independently on multiple molecules. However, damage to a single cell’s genome can affect a large amount (25%; single-stranded error) of the initial template. In an idealized MDA process, MDA replicates all four initial strands (two molecules) of DNA to produce eight strands (four molecules). A random polymerase misincorporation error would affect 1 out of 8 DNA strands, affecting 12.5% of the DNA. **c** Allelic imbalance affects the VAF of both true mutations and artifacts. Over amplification of an allele harboring early single-stranded damage (red) can inflate the artifact to a mutation-like VAF while a true mutation (green) can be reduced to low VAF. **d** Without allelic imbalance, heterozygous SNV VAFs would be tightly distributed around 50% due to random sampling effects. However, allelic imbalance causes VAFs in scDNA-seq to be significantly over-dispersed symmetrically around 50%

The genome of a single cell must first be extracted and extensively amplified to produce sufficient DNA for sequencing on standard high-throughput sequencing platforms. Of the handful of whole-genome amplification (WGA) protocols currently available^5^, multiple displacement amplification (MDA) is presently considered the most suitable for genome-wide detection of single nucleotide variants (SNVs) and short insertions and deletions due its ability to amplify the majority of a human genome with a high-fidelity polymerase^6,7^. However, MDA is a non-linear amplification process and therefore suffers from non-uniform amplification of the genome, creating high variability in sequencing depth along the genome. The same non-uniform amplification process can also cause differences in amplification between homologous copies of the same DNA because they are amplified essentially independently^8^ (Fig. 1a). In human cells, for example, the maternal copy of a gene can be amplified to a different level than the paternal copy, leading to a large disparity in the number of sequencing reads generated from each allele. This allelic imbalance is common in MDA-amplified DNA libraries and substantially complicates the identification of somatic mutations—which appear as heterozygous variants—in scDNA-seq data.

In scDNA-seq applications aiming to identify somatic SNVs, computational filtering of physical artifacts that arise spontaneously during cell lysis, DNA extraction, library preparation, and WGA is essential, as the number of artifacts can vastly exceed the number of true somatic mutations and obscure biologically relevant signals. Artifacts occurring before amplification or in the early stages of amplification could affect a substantial fraction of the few DNA copies at any genomic locus (Fig. 1b). Subsequently, allelic imbalance can further confound detection of such artifacts by over-amplifying the artifact-harboring allele relative to its homolog and can make true mutations harder to detect by under-amplifying the mutation-harboring allele (Fig. 1c). As a result, variant allele fraction (VAF)—the fraction of sequencing reads supporting a heterozygous variant—may deviate substantially from the expected ~50% and is not as informative as it is in bulk sequencing for distinguishing artifacts from true mutations. The effect of allelic imbalance on VAF is often substantial and is evident by examining the VAFs of known heterozygous SNPs in single-cell data (Fig. 1d). Because the VAF of true heterozygous variants in single-cell data should follow the fraction of DNA amplicons from the variant-harboring allele while artifacts do not, the observed VAF of a putative mutation must be appraised considering the allelic-specific amplification balance at that position.

To properly evaluate VAFs in the context of genotyping, we developed a spatial model to estimate allele-specific amplification balance (AB) at any genomic locus. A genome-wide AB curve is constructed by measuring AB at a large set of heterozygous SNPs (obtained prior to scDNA-seq) and inferring AB in the intervening regions. Specifically, the model considers how AB measurements at heterozygous SNPs (hSNPs) correlate with AB away from hSNPs and how to properly combine information from multiple hSNPs in a large neighborhood. This approach proves particularly fruitful in MDA-amplified libraries in which the characteristically long amplicon lengths (typically ~5–10 kb) cause the AB to change relatively slowly along the genome.

The AB model enabled the development of a novel somatic SNV genotyper called SCAN-SNV (single-cell analysis of SNVs). SCAN-SNV removes scDNA-seq artifacts by requiring candidate sSNV VAFs to both match the estimated local AB and not match VAFs consistent with common scDNA-seq artifacts. SCAN-SNV also employs a novel method to estimate artifact burden and an upper limit on the number of true somatic mutations prior to genotyping, which helps to address situations in which the artefactual mutations substantially outnumber somatic mutations. Standard SNV genotypers designed for bulk data have been shown to perform very poorly when applied to single cells, primarily by calling a large number of artifacts as true mutations^9,10^. Our comparative analyses show that SCAN-SNV substantially outperforms both Monovar^9^ and SCcaller^10^, with a >3-fold decrease in false discovery rate while maintaining similar sensitivity.

## Results

### Distinguishing artifacts from mutations using allele balance

Below, we demonstrate how allelic imbalance can lead to false positive (FP) variant calls in practice and how estimating AB can help to avoid such erroneous calls. We also show how to approximate the prevalence of single-cell artifacts and bound the somatic mutation rate prior to genotyping, which can help to control the false discovery rate (FDR). Finally, we integrate these ideas into our sSNV genotyper SCAN-SNV and apply it to a scDNA-seq benchmarking data set.

**Fig. 2:**
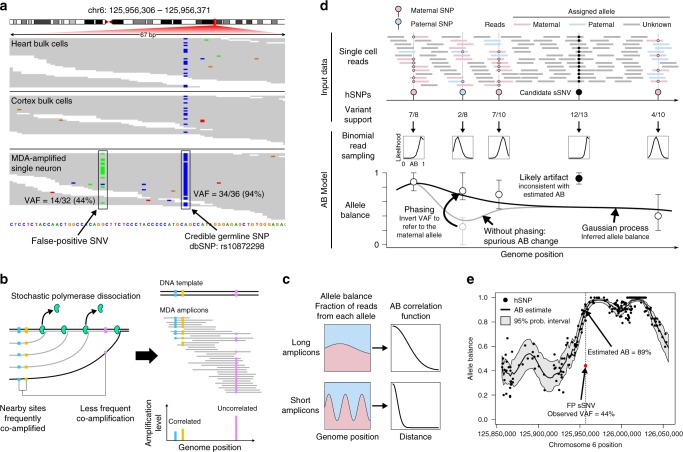
The allele balance model helps to identify single cell artifacts. **a** A single cell artifact (left, green) attains high VAF (44%). The region is affected by allelic imbalance as evidenced by the hSNP with VAF 94% (right, blue). The candidate sSNV should present with VAF ≈ 6% or VAF ≈ 94%. **b** MDA polymerase (green) randomly dissociates from the template DNA belonging to one allele (black), creating amplicons (gray) of various lengths. Nearby sites are highly likely to be amplified by the same polymerase, but the probability decreases for more distant sites. This creates a correlation in allele-specific amplification levels between nearby sites (blue, orange). The process occurs independently on both homologous alleles, leading to a stable allele balance in a small genomic locus. **c** Long amplicons cause allele-specific read depths (blue, paternal allele; pink, maternal allele) to change more slowly along the genome. When each allele is more stable, so is the allelic balance. The AB correlation function quantifies allele balance stability. **d** Illustration of AB modeling and estimation. Reads at hSNPs can be assigned to alleles based on whether they contain reference- or variant-supporting bases. This allows allele-specific depth, and therefore AB, to be estimated at the hSNP. AB outside of hSNP loci is inferred (thick black line) using a Gaussian process parameterized by the AB correlation function. A binomial read sampling model determines how closely the inferred AB curve should follow the noisy hSNP measurements (error bars: 95% confidence intervals). Phasing hSNPs allows the paternal SNP (blue) VAF to be adjusted to (1 – VAF) to be consistent with the surrounding maternal SNPs, which is necessary to produce long-range allele balance estimates. The shown candidate sSNV, despite achieving very high VAF, is likely an error since it does not match the local amplification balance. **e** The AB model applied to a 200 kb window around the candidate sSNV shown in (**a**). The artifact (red) at VAF = 44% is highly inconsistent with the model’s estimated AB of 89% (black line) and falls well outside of the 95% probability interval (gray envelope)

In general, candidate mutations supported by a high fraction of sequencing reads are less likely to be artifacts than low VAF candidates. However, even high VAF candidates must be carefully examined in scDNA-seq data because a considerable number of single-cell artifacts attain high VAF due to allelic imbalance. In Fig. 2a, we provide an example of a high VAF (44%) artifact present in a single neuron^3^. Although the high VAF is promising initially, the nearby hSNP rs10872298 (a well-characterized polymorphism, also found in bulk samples of the same individuals) is supported by 94% of reads, indicating that the region is severely affected by allelic imbalance. Therefore, acceptable candidate mutation VAFs should be similar to either 6 or 94% since all reads from the mutated allele should support the sSNV. On the other hand, a single-stranded, pre-amplification artifact on the over-amplified allele would be supported by half of reads from that allele on average (94%/2 = 47%, Fig. 1c), which closely matches the observed VAF. This suggests the candidate sSNV should be rejected despite its high VAF and demonstrates the utility of AB in evaluating the fraction of reads supporting a mutation.

However, this simple method to estimate AB and apply it to candidate sSNVs is problematic. First, although the hSNP in Fig. 2a is very close (20 bp) to the candidate sSNV, the average distance between credible hSNPs in the data analyzed here is ~1500 bp. Thus, a robust estimate of AB should account for the distance from the neighboring hSNPs. Second, VAF at an hSNP can be a noisy estimator of AB at typical whole-genome sequencing depths. For example, an approximate 95% AB confidence interval in Fig. 2a ranges from 86 to 100% and would widen at lower sequencing depth. It is therefore important to model this noise and to incorporate data from as many informative hSNPs as possible to increase the precision of the estimate.

### Estimating allele balance genome-wide

To address these issues, we developed a genome-wide AB model that conceptualizes AB as a smooth curve along the genome representing the fraction of DNA amplicons derived from one (arbitrarily chosen) of the two alleles. The model is based on the principle that AB measured at an hSNP is most applicable in the immediate vicinity and becomes increasingly less informative at more distant loci. This happens because amplification products covering the hSNP site also cover nearby genomic loci with high probability, leading to similar levels of amplification and therefore similar AB (Fig. 2b). Sites further away from the hSNP are less likely to be amplified together, allowing for different amplification levels and possibly different AB. Therefore, the distance at which AB measurements remain correlated depends on the distribution of amplicon sizes, with larger sizes corresponding to slowly changing AB curves (Fig. 2c). Average amplicon sizes for MDA can range from ~5 to 10 kb but can vary substantially between samples even when the same amplification protocol is followed. We employ a Gaussian process to formally model how AB correlation decays as a function of distance and to combine information from multiple hSNPs in a statistically principled way for prediction of the AB at any genomic position (see Methods section).

Figure 2d illustrates how the model is trained and applied. The input data are the locations of phased, credible hSNPs from an external source (e.g., matched bulk sequencing data or a SNP database) and the number of reads supporting the variant and reference alleles at each hSNP in the single cell. To determine the likely AB values at the hSNP, read counts—not VAFs—are used in a binomial model to account for random fluctuations due read sampling. If adjacent hSNPs are located on opposite alleles, a sudden, but spurious, change in AB might appear (Fig. 2d, gray line), and this could severely impede learning the AB correlation properties. We solve this by phasing hSNPs and standardizing all AB measurements to an arbitrary but consistent allele. The AB model is trained by choosing the AB correlation function that maximizes the model likelihood over phased hSNPs. To infer AB at a site of interest, the Gaussian process produces a Bayesian posterior AB distribution using the learned correlation function to automatically find and combine information from all informative hSNPs. To evaluate candidate sSNV mutations, we built three statistical tests based on AB predictions: (1) an allele balance consistency (ABC) test to determine whether a candidate is consistent with the local AB; (2) a test for pre-amplification artifacts which looks for candidate mutations occurring on roughly half of reads from either allele; and (3) a test for early amplification artifacts which, under an ideal amplification process, occur on one quarter of reads from either allele (see Methods).

We applied the AB model to a 200 kb window around the locus in Fig. 2a. As shown in Fig. 2e, the FP sSNV (red) is visually inconsistent with the AB in its region, lying well outside of the 95% confidence interval (gray envelope). The candidate sSNV is rejected by the ABC test with *p*-value 1.8 × 10^−6^ and is instead consistent with a pre-amplification artifact (*p*-value = 0.54).

Despite the improved ability to reject scDNA-seq artifacts made possible by our model, it remains difficult to predict AB in large genomic regions devoid of hSNPs. The model is also susceptible to errors in hSNP phasing in which the variant is assigned to the incorrect allele. However, phasing errors are infrequent and only produce a noticeable shift in AB in imbalanced regions. It is important to note that the model does not account for copy number changes or structural variations, which can alter VAF and affect the apparent AB, and assumes that the two strands of DNA from a single allele are equally amplified. If a single DNA strand went completely unamplified, then physical scDNA-seq artifacts could present at the same VAF as true mutations. Finally, in regions with specific AB values, VAF alone may not be sufficient to distinguish artifacts from true sSNVs regardless of sequencing depth. For example, when the allele balance is 2:1, both a true sSNV on the under-amplified allele and a pre-amplification artifact on the over-amplified allele would attain VAF = 1/3 on average. SCAN-SNV rejects both true sSNVs and artifacts in such regions by the pre-amplification artifact test; a similar argument applies to regions with 4:1 allele balance and the amplification artifact test. As a result, SCAN-SNV rarely has power to call SNVs with VAF < ~40% at typical whole-genome sequencing depths of 30×.

### Tuning calling thresholds to account for artifact prevalence

**Fig. 3:**
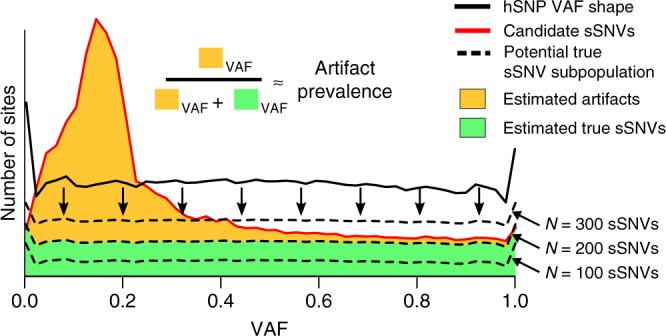
SCAN-SNV FDR tuning strategy. Somatic SNVs and hSNPs are supported by 50% of DNA prior to amplification in single cells. The shapes of VAF distributions for the two mutation types should be similar because both are equally affected by allelic imbalance, but artifacts in the candidate sSNV set (red line) usually create an enrichment at low VAF compared with hSNPs (black line). VAFs for the unknown number of true mutation among candidate sSNVs (green area) should be distributed similarly to hSNPs. Potential values for the total number of true sSNVs *N* (dashed lines) can be evaluated by first distributing the *N* mutations according to the hSNP VAFs and then ensuring the predicted numbers of sSNVs at each VAF do not exceed the number of candidates at that VAF. The largest such *N* provides an upper bound on the number of somatic mutations. Given *N*, a lower bound on the fraction of artifacts amongst sSNVs at any VAF can be estimated

For the statistical tests derived from the AB model, *p*-value thresholds for calling must be set. Because scDNA-seq artifacts tend to be enriched at low VAF (Fig. 3, red curve), we reasoned that increasing the stringency of *p*-value thresholds for low VAF candidate mutations would increase accuracy. In particular, a *p*-value threshold *α* can be related to the false discovery rate by

1 $${\mathrm{FDR}} \approx \frac{{\alpha N_{\mathrm{A}}}}{{\alpha N_{\mathrm{A}} + \left( {1 - \beta } \right)N_{\mathrm{T}}}},$$

where *β* is the type II error rate resulting from the choice of *α*; *N*_T_, and *N*_A_ are the number of true mutations and artifacts in the candidate sSNV set, respectively. Given suitable estimators of *N*_T_ and *N*_A_, it would be possible to adjust *p*-value thresholds *α* to target a user-supplied FDR.

We therefore developed a method to estimate an upper bound on *N*_T_ by exploiting the fact that hSNPs and somatic SNVs are present on 50% of DNA molecules prior to amplification and are equally affected by allelic imbalance. Under the assumption that somatic SNVs occur in diploid regions and are approximately uniformly scattered across the genome, VAF distributions for hSNPs and sSNVs should be similarly shaped. The unknown subset of true sSNVs resides in the larger set of candidate sSNVs and should form a VAF distribution similar to that of hSNPs (Fig. 3). We employ a heuristic to find the largest number of true sSNVs *N*_T_ that fits beneath the candidate VAF distribution when distributed according to the hSNP VAFs (see Methods). *N*_T_ can be combined with the hSNP VAF distribution to estimate the number of true mutations and artifacts at each specific VAF, allowing the determination of VAF-specific *p*-value thresholds corresponding to the desired FDR (see Methods). This does not constitute formal FDR control, but rather provides a rough guide to increasing stringency for sSNV classes that are contaminated by a large artifact burden.

### SCAN-SNV: genotyping sSNVs in MDA-amplified single cells

**Fig. 4:**
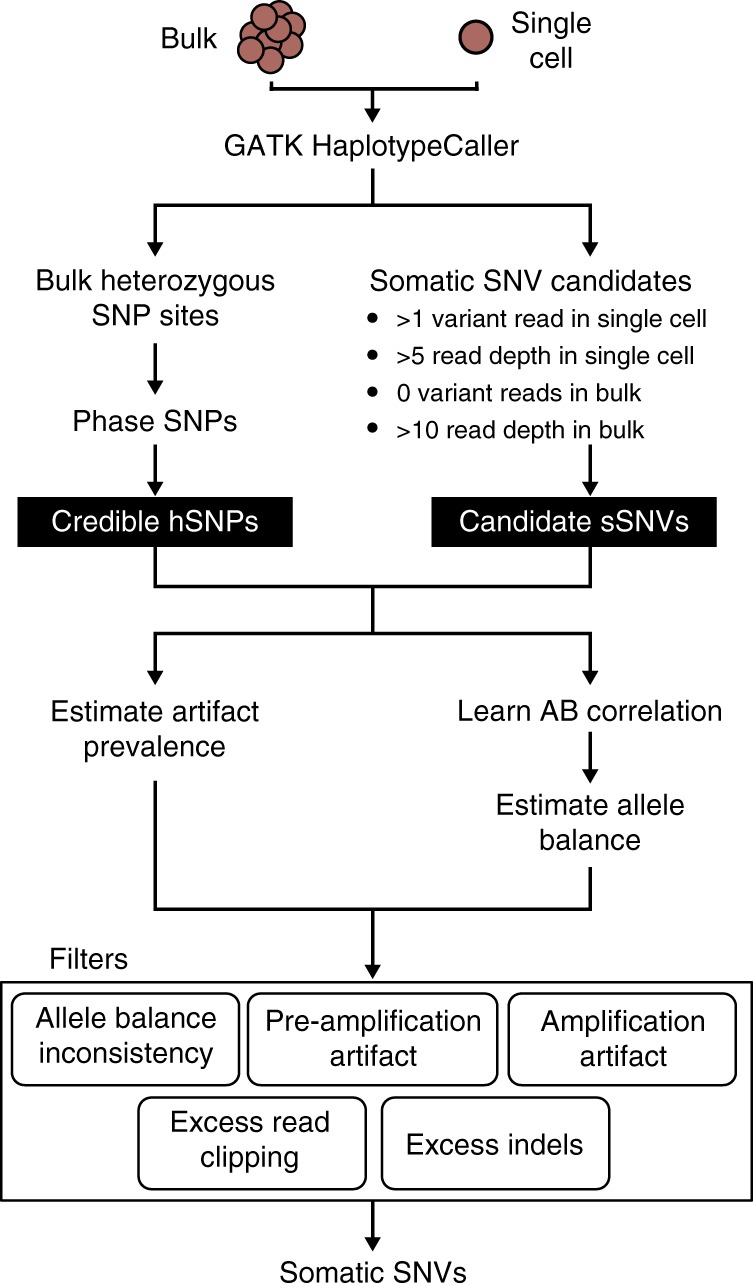
SCAN-SNV workflow. GATK HaplotypeCaller determines sites with non-reference evidence and discovers germline or clonal hSNPs from bulk. Phased hSNPs serve as a training set to learn AB correlation patterns, predict AB at candidate sSNV loci and estimate artifact prevalence. Only candidate sSNVs passing all filters are reported as putative mutations

We combined the AB model and automatic threshold tuning to create SCAN-SNV (Fig. 4). An overview of the method follows. First, GATK^11^ HaplotypeCaller is jointly applied to both single cell and bulk sequencing data to generate a genome-wide list of non-reference sites and their respective variant and reference read counts. The calls in the matched bulk data are taken as hSNPs, which are then phased by SHAPEIT2^12^ and used to train the AB model.

Somatic SNV genotyping begins by defining a set of candidate sSNVs using the lenient read support requirements shown in Fig. 4. Their VAF distribution is then compared with the phased hSNP VAF distribution to estimate the artifact burden and somatic mutation rate. The AB model’s correlation function is fit separately for each chromosome by inspecting single-cell reads at phased hSNPs. Finally, AB is estimated at every candidate sSNV and several filters designed to remove single cell artifacts are applied. Primary filters are based on the AB model and include the three previously described statistical tests. The artifact statistical tests are tuned at every candidate site to obey a user-specified target false discovery rate given the estimates of artifact prevalence. Secondary filters remove candidate sSNVs covered by an excessive number of reads containing indels or clipped reads compared with reads at the same locus in the bulk sample. Candidate sSNVs passing all filters are emitted as putative somatic SNVs. Finally, when multiple related single cells are sequenced, an optional joint calling mode which relies on repeated observations of allele balance consistency across samples can be applied (see Methods).

### Assessing the performance of SCAN-SNV

It is difficult to validate somatic SNVs in scDNA-seq data because the genome of a single cell is consumed during WGA, i.e., DNA extraction and amplification cannot be replicated to identify artifacts. Validation of putative sSNVs by deep sequencing of excess amplified DNA not used for initial sequencing can be confounded by the artifacts that were introduced in the amplified DNA; validation in the original tissue by very high-depth amplicon sequencing is possible^3^ but only for clonal sSNVs with VAFs that are not too small. We therefore assessed SCAN-SNV and other callers using two approaches: a synthetic data set and a kindred cell system.

### Performance assessment on simulated data

We first assessed SCAN-SNV using a synthetic diploid (SD) male chromosome X benchmarking data set. Hemizygous male X chromosomes have been previously exploited in single-cell studies for estimating FDR^3^, utilizing the fact all true mutations on the hemizygous chromosome should have VAF close to 100%. However, a hemizygous X chromosome cannot reproduce the challenge of calling variants on a diploid chromosome because the AB should be either 0 or 1 along most of the chromosome. We therefore created synthetic diploid (SDs) X chromosomes by mixing chromosome X reads from the single cells of two different male donors with spiked-in mutations serving as a truth set. Unlike purely simulated approaches, the SD X chromosomes retain the patterns and characteristics of true MDA artifacts. Disadvantages of SDs include the fact that data from different amplifications are combined (although the same amplification protocol was followed) and that chromosome X has a lower SNP density than autosomes, which can disadvantage callers that depend on local hSNPs (such as SCAN-SNV and SCcaller).

**Fig. 5:**
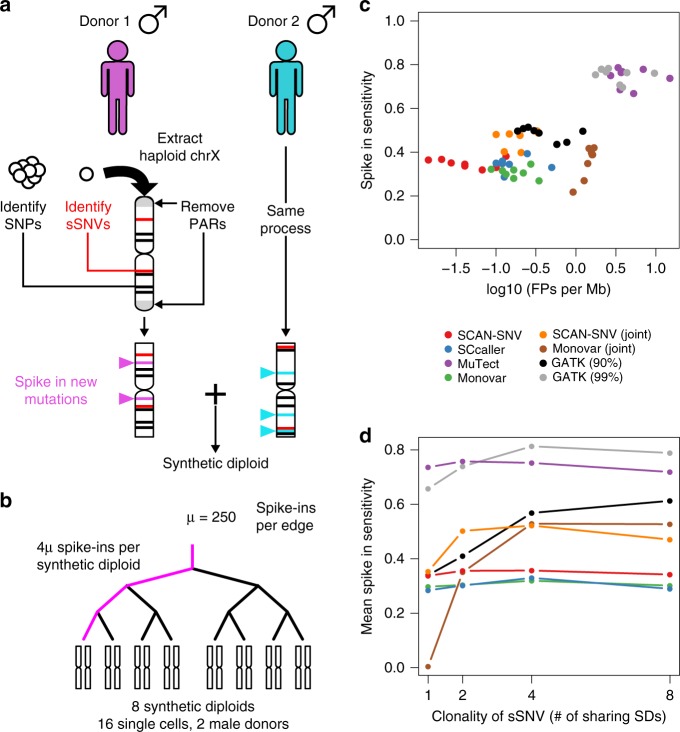
Synthetic diploid construction and performance. **a** Synthetic diploid X chromosomes (chrX) are generated by merging chrX reads from single cells of two male donors. After chrX reads are extracted, pseudoautosomal regions (PARs) are removed and SNPs and sSNVs are identified on the hemizygous regions of chrX. New, randomly placed somatic mutations are spiked into the reads. This process is repeated for a second male donor and the two sets of reads are merged to create a synthetic diploid (SD). **b** Each SD contains 1000 spike-in mutations with 750 mutations shared by other SDs. For example, 250 spike-ins are shared by all SDs, another 250 are shared by four SDs and so on. **c** Each point represents genotyper performance on one SD. Only spike-in mutations are used to compute sensitivity. False positive rate is the number of FPs divided by the number of non-PAR megabases on chrX. **d** Spike-in sensitivity for private spike-ins (clonality = 1), spike-ins shared by two samples (clonality = 2), etc

Briefly, reads from single cells were processed prior to mixing by first identifying endogenous SNPs and sSNVs and then spiking in 500 randomly placed mutations using BAMSurgeon^13^ (Fig. 5a, see Methods). Processed reads from 16 single cells from two male donors^4^ were then downsampled and merged to create eight SDs with mean 30× depth (Supplementary Table 1). A matching synthetic diploid bulk sample was created by mixing bulk data for the two male donors. To allow benchmarking of multi-sample genotypers, some of the spike-in mutations were shared across multiple SDs according to the phylogeny depicted in Fig. 5b.

SCAN-SNV achieved the lowest false positive rate (FPR), improving by ~3-fold over SCcaller and Monovar in single-sample mode and by ~30-fold over Monovar in joint mode (Fig. 5c). When both shared and private spike-in mutations were considered, sensitivity was very similar across the four single cell genotypers (mean sensitivity 30–35%) with Monovar in joint mode achieving the highest average recall. Notably, Monovar’s allowance for up to one mutation supporting read in the matched normal sample and lack of dbSNP filtration may lead to higher sensitivity at the cost of increased FPR. The highest sensitivity was attained by MuTect^14^ and GATK as expected, but the number of FPs committed by these callers renders them impractical for studies where low- to moderate mutation burdens are expected.

To better assess multi-sample genotypers, sensitivity was also assessed for shared spike-in mutations (Fig. 5d). As expected, single-sample callers do not show increased sensitivity for mutations supported by multiple samples. SCAN-SNV’s joint mode, which calls mutations with good allele balance consistency across several samples (see Methods), committed 10-fold fewer errors than Monovar in joint mode and matched or exceeded Monovar’s sensitivity for sSNVs shared by fewer than eight samples. Although GATK HaplotypeCaller (90% sensitivity target) performed well, the difference between its target (90%) and actual performance (40–60% spike-in sensitivity) suggests that it would be difficult to tune in practice.

### Performance assessment using a kindred cell system

We also validate the performance of our method using a kindred cell system, in which one amplifies and sequences two or more very closely related single cells (e.g., separated by <5 cell divisions) so that essentially all somatic mutations are shared. This enables discrimination between true mutations, which are shared between cells, from spontaneous artifacts, which are unlikely to recur at the same positions in multiple amplifications.

**Fig. 6:**
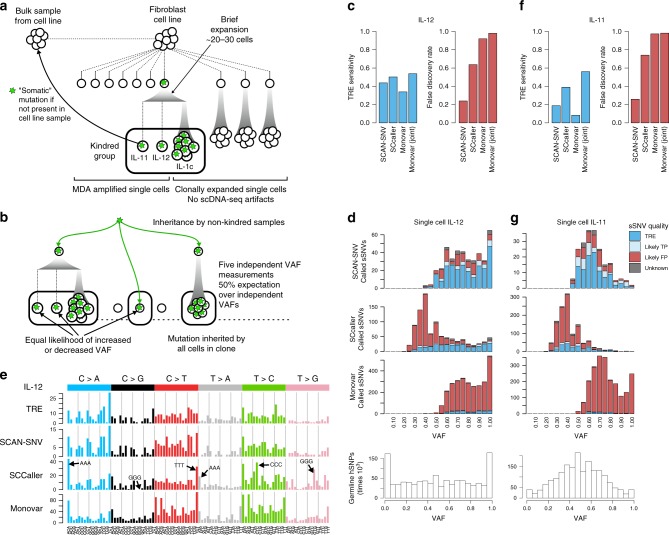
SCAN-SNV performance assessed by a kindred cell system. **a** Twelve single cell-derived samples from a human fibroblast cell line^10^. Somatic mutations are defined as mutations not observed in the cell line bulk. A kindred cell system comprising three very closely related samples mimics biological replicates of single cells and enables assessment. True mutations (green stars) are likely to be supported by several kindred samples; scDNA-seq artifacts, however, should be private. **b** sSNVs in kindred samples with non-kindred support may be subclonal sSNVs. If true, each single cell sample inheriting the subclonal sSNV provides an independent VAF measurement. For true subclonal mutations, the average VAF over many samples should be ~50%. **c** Single cell genotyper performance on kindred cell IL-12. TRE sensitivity, percent of triple exclusive sites (TREs) recovered; FDR, fraction of total calls classified as likely FPs. **d** sSNV calls for both genotypers are binned by VAF and classified as either TRE, likely TP or likely FP based on which of the 13 samples contain read support for the mutation. Triple exclusive (TRE) sites are high-quality sites supported by all kindred samples and no other samples. Monovar is run in single-sample mode. (Bottom) hSNP VAFs provide a reference distribution for sSNVs. **e** Trinucleotide mutation signatures for TREs and genotyper calls in kindred cell IL-12. Monovar is run in single-sample mode. Arrows indicate mutation contexts which create homopolymers. **f** Same as (**c**) for kindred cell IL-11. **g** Same as (**d**) for kindred cell IL-11. The VAF distribution of both hSNPs and TRE sSNVs is very different from IL-12, suggesting substantial differences in amplification or cell quality

We therefore assessed the performance of SCAN-SNV with a previously published kindred system^10^ which contains two MDA-amplified single cells (IL-11 and IL-12) and an unamplified, single cell-derived clone (IL-1c). Their experimental design (Fig. 6a) also contains six non-kindred MDA-amplified single cells, three unamplified single cell-derived clones and an unamplified bulk sample that serves the role of a matched normal tissue.

In the previous analysis of the kindred group^10^, sSNVs were considered validated if they were called in a kindred single cell and supported by 4 or more reads in the unamplified kindred clone (IL-1c). This analysis relies solely on the unamplified cell line bulk to prevent the validation of recurrent artifacts; however, this strategy is questionable because the unamplified cell line bulk cannot account for recurrent artifacts caused by amplification. (We also found systematic alignment differences because the cell line bulk and kindred group samples were sequenced using different read lengths, 100 bp vs. 250 bp).

We undertook a more comprehensive approach to validation, taking advantage of the information provided by the additional nine single cell-derived samples (mean total coverage ~264x) from the same cell line. We integrated calls from both GATK and samtools^15^ to classify sSNVs as likely TPs, likely FPs, and unknown, depending on the patterns of shared support across all 12 single cell-derived samples (see Methods, Supplementary Table 2). Briefly, likely TP sSNVs must be supported by at least 50 total reads across all 13 samples, a minimum number of mutation-harboring reads across the kindred group (number determined by a mock analysis of non-kindred samples, see Methods, Supplementary Fig. 1) and by at least two kindred samples. Furthermore, to exclude recurrent artifacts, they must not be supported any of the remaining non-kindred samples. Likely TP sSNVs supported by all three kindred samples exclusively (‘triple exclusive’; TRE) are the highest confidence somatic mutations and are used to assess genotyper sensitivity. The remaining sSNVs, which are either supported by one kindred sample (singletons) or by samples outside of the kindred group, are classified as likely FPs (see Methods, Supplementary Table 2). Most singletons are physical scDNA-seq artifacts and the small number of true mutations private to a single cell cannot be validated by the kindred system. sSNVs supported by out-of-kindred group samples (which could be true, subclonal mutations) also appear to be primarily FPs, since they occur with reproducibly low VAFs across samples (mean VAF = 16%) when true somatic mutations inherited by multiple single-cell samples should not have consistently low VAFs (Fig. 6b). We therefore assessed FDR by the fraction of likely FP sSNV calls over all positive calls.

SCAN-SNV called 397 sSNVs in the kindred single cell IL-12 with an estimated FDR of 24% and an estimated sensitivity of 44% (Fig. 6c). As previously discussed, sSNVs and hSNPs should have similar VAF distributions. Indeed, above VAF = 50%, mutations called by SCAN-SNV follow the hSNP distribution (Fig. 6d bottom) reasonably well. However, there were very few SCAN-SNV calls below VAF = 50%, due to reduced power to distinguish low VAF true mutations from artifacts. Power to resolve the two cases depends on sequencing depth, and we anticipate that SCAN-SNV is capable of detecting more low VAF mutations in samples with higher depth.

SCAN-SNV performs favorably compared with SCcaller, which showed slightly increased sSNV sensitivity (44 vs. 50% of TREs recovered) but produced a call set highly enriched for likely FPs, leading to a ~3-fold increase in estimated FDR (24 vs. 64%). Independent of our assessment scheme, the large number of likely FPs called by SCcaller is evident by a considerable skew toward low VAF sSNVs that is not observed for hSNPs (Fig. 6d, middle compared with bottom). Monovar achieved the highest sensitivity when run jointly on all eight single cells (54%) but was comparatively low when run on a single sample (34%). However, Monovar produced a very large number of FP calls in both modes (FDR: 92% single, 98% joint).

As shown in Fig. 6e, mutation signatures based on trinucleotide frequencies^16^ for TRE sSNVs are characterized by the pattern of C > A mutations similar to a previously observed signature associated with cell culture^17^. This is consistent with the anticipated mutagenic process acting in these samples, which were all taken from a human fibroblast cell line. The mutational pattern for SCAN-SNV calls in IL-12 was concordant with that of TRE sSNVs. However, SCcaller sSNVs for IL-12 were generally enriched for T > C and T > G mutations and contained pronounced peaks for mutations that would create homopolymer runs (e.g., ACA > AAA, GCG > GGG, etc.). We speculate that these peaks may correspond to artefactual calls in repetitive regions such as microsatellites, where accurate alignment is particularly difficult. Monovar calls contained peaks at all ANA and TNT contexts (first and last columns of each color group) and shared SCcaller’s enrichment for T > C and T > G mutations in general. Monovar in multi-sample mode shared these characteristics and also contained a striking peak for TCA > TAA mutations, which may point to a reason for the increase in FPR (Supplementary Fig. 2).

### Detection of atypical MDA amplification

SCAN-SNV was also applied to the other kindred single cell, IL-11. In this cell, 196 sSNVs were called with an estimated FDR of 26% and TRE sensitivity of 19% (Fig. 6f). SCAN-SNV again compared favorably with SCcaller in FDR, attaining a ~3-fold reduction (26 vs. 74%), similar to IL-12. But it showed marked reduction in sSNV sensitivity compared with SCcaller (19 vs. 39%) and its own performance on IL-12 (explained below). Monovar’s single-sample sensitivity also suffered (8 vs. 34%), but its sensitivity in joint mode was unaffected (56 vs. 54%). Again, however, Monovar’s FDR remained exceptionally high (97–99%).

**Fig. 7:**
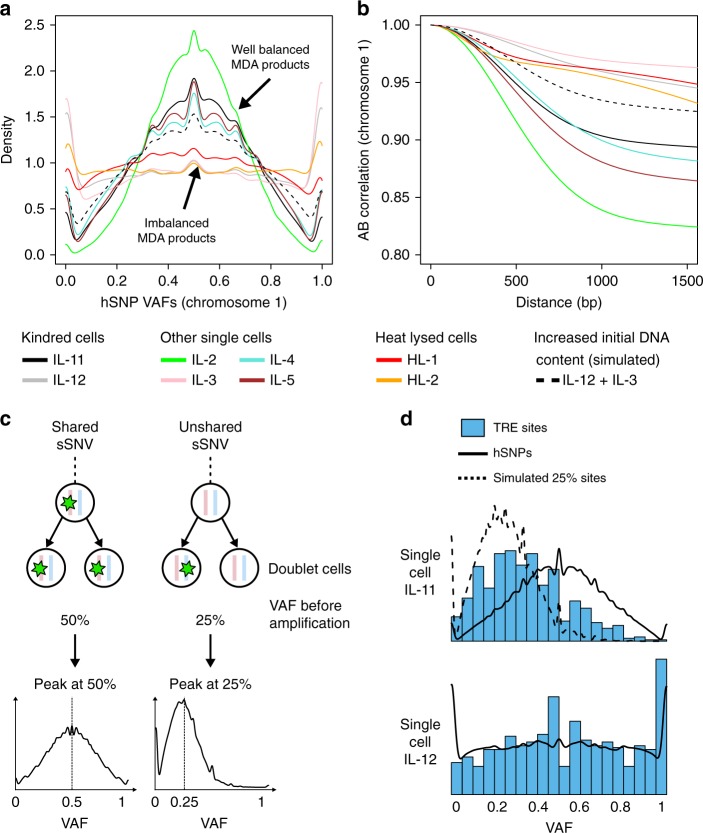
SCAN-SNV recognizes atypical MDA amplification and reveals an accidental doublet. **a** Chromosome 1 hSNP VAF distributions for eight MDA-amplified single cells fall into one of two classes. Distributions concentrated near half are well-balanced (IL-11-like) whereas flat distribution with peaks near 0 or 1 are imbalanced (IL-12-like). Dashed line: a simulation of a single cell with increased initial DNA content resembles the well-balanced class. **b** Chromosome 1 AB correlation functions for each MDA-amplified single cell. Well-balanced products are less correlated at short distance than imbalanced products. Dashed line: the increased initial DNA content simulation resembles well-balanced samples. **c** When two cells are amplified instead of one (an accidental doublet), some somatic mutations will be shared by both cells while others will not. Unshared mutations are supported by only 25% of DNA before amplification. If there are enough unshared somatic SNVs, a doublet sSNV VAF distribution will resemble a mixture of peaks near 25 and 50%. A similar pattern is not expected for a G2/M phase cell. **d** sSNV VAF distributions are estimated using triple exclusive (TRE) sSNVs (blue). hSNPs show the correct VAF distribution for shared sSNVs and the expectation for unshared sSNVs (dashed line) is obtained by downsampling hSNPs. The peak near VAF = 25% for IL-11 indicates a large number of unshared variants, identifying the sample as an accidental doublet

Despite the fact that both kindred cells were very closely related and processed according to the same protocols, SCAN-SNV immediately revealed two clear differences in the quality of the two sequencing libraries that may explain the change in genotyping accuracy. First, hSNPs in IL-11 were considerably more concentrated around VAF = 50% than in IL-12 (Fig. 6d, g, bottom). Second, AB correlation decayed more quickly at short distances in IL-11 than in IL-12. hSNP VAF distributions and AB correlation functions for the six additional, non-kindred MDA-amplified single cells also closely matched the VAF and AB patterns of either IL-11 or IL-12 (Fig. 7a, b), forming two distinct classes: well-balanced MDA products resembling IL-11 and imbalanced products resembling IL-12. The same two classes could also be created by separating single cells on the basis of genome-wide copy number profiles, which were computed for all samples using Ginkgo^18^ (Supplementary Fig. 3). It is natural to assume these differences are caused by random variability in the quality of WGA; however, the observation that samples cluster into two distinct classes rather than varying more continuously is inconsistent with this hypothesis.

### Identification of an accidental doublet

One explanation for the well-balanced MDA products is that the cells had increased DNA content prior to amplification. For example, when amplification begins with two genomes rather than one, the additional copies reduce the variability in read depth and lead to more balanced amplification between alleles—a fact which has been exploited previously^1^. In the present data set, the increase in DNA content could have been due to (i) cells in G2 or early M phase; or (ii) the accidental isolation of two cells rather than one (a doublet). In either scenario, AB correlation should decay more rapidly since each allele is initially represented by two copies which are amplified independently. Indeed, when we simulated this increased DNA content by combining reads from two imbalanced samples (see Methods), its hSNP VAF distributions and AB correlation functions matched those of well-balanced products (Fig. 7a, b, dashed lines).

Further analysis of sSNV VAF distributions revealed that IL-11 is highly likely to be a doublet. Unlike hSNPs, sSNVs may or may not be shared by both cells in the doublet. Unshared sSNVs would be present on 25% of DNA prior to amplification whereas shared sSNVs would be supported by 50% (Fig. 7c). Therefore, a positive indicator for a doublet is a peak at VAF = 25% in sSNV VAFs. We computed sSNV distributions for IL-11 and IL-12 using TREs to avoid genotyper bias (Fig. 7d) and confirmed the doublet hypothesis for IL-11 by observing the characteristic peak at VAF = 25% for IL-11 but not in IL-12.

An accidental doublet explains SCAN-SNV’s reduced sensitivity for IL-11: sSNVs not shared by both cells in the doublet would be filtered because they occur at the same VAF expected by our model of pre-amplification artifacts.

### Performance on single tumor cells

SCAN-SNV was developed with the assumption that all DNA sequences are diploid. When this assumption does not hold, e.g., when sequencing single cells from tumors, it may perform suboptimally. To test its performance in aneuploid cells, we applied it and other callers to four single estrogen receptor positive breast cancer (ERBC) cells that harbor several chromosome-level copy number changes^1^. This data set was not sufficient to construct a truth set, so callers were judged by the fraction of sSNVs that were supported either in bulk sequencing of the same ERBC cancer cell population or in multiple single cells (clonal support). SCAN-SNV’s calls were most frequently clonally supported (55% clonal support, 955 total sSNV calls, Supplementary Fig. 4a) whereas other callers either produced relatively few calls (SCcaller, 35% clonal support, 532 calls) or recovered more clonally supported sSNVs at the cost of many suspect calls (Monovar and MuTect, 5–7% clonal support and 18,049–66,008 calls) (Supplementary Fig. 4b). Clonal support rates for SCAN-SNV calls were computed separately for haploid, diploid and triploid chromosomes to explore the effects of violating the diploid assumption (Supplementary Fig. 4c). Haploid chromosomes showed slightly increased clonal support (mean 60%) compared with diploid and triploid chromosomes (48% and 52%, respectively). Increased performance on haploid chromosomes is expected since true mutations will have VAFs near 100% while the similar performance for diploids and triploids provides evidence that SCAN-SNV can gracefully handle some violations of its diploid assumption. We caution that chromosome-level copy number changes are mild violations of the diploid assumption and that performance may degrade under more severe violations such as chromothripsis, structural variants, and sub-chromosomal copy number variants (CNVs).

## Discussion

Analysis of scDNA-seq data presents many challenges. For sSNV detection, they include filtration of high VAF artifacts caused by cell lysis, whole-genome amplification and recurrent analysis artifacts such as read misalignments. Our analyses suggest that most single-cell genotypers have similar sensitivities for detecting somatic SNVs in diploid cells but commit false positive errors at substantially different rates. SCAN-SNV achieves unprecedented specificity by estimating allelic imbalance and only calling mutations when sufficient power is available to avoid false positives. The efficacy of our methodology was further confirmed by the unanticipated detection of a doublet.

In particular, SCAN-SNV outperforms SCcaller, the only published genotyper for non-clonal SNVs in scDNA-seq data. The only other genotyper designed for single cells is Monovar, but it is intended for joint calling across multiple cells, and its authors specifically advise users to remove private calls. Although SCcaller also attempts to remove artifacts by estimating AB from hSNPs, the lower false discovery rate (~3-fold reduction) for SCAN-SNV stems from major improvements to both the method of estimating AB and the genotyping procedure: (i) SCcaller estimates AB by computing weighted moving averages of hSNP VAFs over a fixed size sliding window, but these weights and window sizes are not learned from real MDA data, not fit to individual samples, and do not account for sequencing depth (i.e., a VAF = 50% hSNP covered by 10 reads will be treated the same as a VAF = 50% hSNP covered by 100 reads, despite the fact that the higher depth SNP contains more information); (ii) SCcaller does not phase the VAFs used in the moving average and limits its model to estimating the fraction of DNA from the most amplified allele; (iii) SCcaller does not provide probability intervals for AB estimates; (iv) SCcaller’s genotyping procedure does not include a model for pre-amplification scDNA-seq errors, which are expected to attain the highest VAFs; (v) SCcaller only provides parameters to control sensitivity to germline hSNPs rather than the FDR, which is more critical in scDNA-seq applications where true mutations are often scarce compared with artifacts; (vi) finally, SCcaller does not include power calculations to determine which conditions (e.g., read depth, candidate sSNV VAF, AB) are necessary to reliably distinguish mutations from artifacts.

We anticipate that our genotyping method, which primarily depends on VAF, can be extended and improved through integration with non-VAF information such as physical read linkage^19^. The linkage approach can evaluate sSNV candidates that reside on the same sequencing read as nearby hSNPs by computing the apparent number of haplotypes at the locus. scDNA-seq artifacts usually occur on a subset of reads from one allele, creating evidence for three haplotypes at the locus when a single cell should contain only two. Although linkage can resolve sSNVs intractable to VAF analysis, its main disadvantage is that it applies only to a relatively small fraction of the genome (~20–25%).

Further, our AB model can also aid detection of CNVs in scDNA-seq data. AB values in regions affected by CNVs will not oscillate around AB = 1/2 as they would in diploid regions, e.g., AB in a single-copy gain region should oscillate around 1/3 or 2/3 whereas AB in a single-copy loss would tend toward AB = 0 or 1. To distinguish CNVs from artefactual shifts in AB caused by imbalanced amplification, one could determine the typical size of such AB ≠ 1/2 regions resulting from allelic imbalance from our model. Candidate CNVs significantly longer than allelic imbalance segments are more likely to be true events.

In summary, our model of AB, approach to single-cell sSNV genotyping and benchmarking processes are highly useful in their current form and will likely provide a solid foundation for future improvements to single-cell genotyping. The AB framework developed here is likely to be helpful for detecting other point-like mutation types (indels) or mutations with point-like features (i.e., breakpoints for structural rearrangements).

## Methods

### hSNP training set construction

SCAN-SNV determines hSNPs by joint application of GATK HaplotypeCaller to all single cell and bulk BAMs and subsequently applying SHAPEIT2 to autosomal, biallelic, single nucleotide non-reference sites called in the bulk sample. The 1000 Genomes Phase 3 integrated haplotype panel dated October 2014 is used for analyses in this report. Only heterozygous SNPs that were successfully phased by this process were treated as credible hSNPs.

### Modeling allele balance

Let *Y*_*i*_, *D*_*i*_, and *X*_*i*_ be the observed number of mutation supporting reads, total reads and genomic position (in base pairs) at locus *i*. We model the allele balance *B*_*i*_ as a latent variable by

2 $$\begin{array}{l}Y_i|D_i,X_i,B_i\sim {\mathrm{Bin}}\left( {D_i,\frac{1}{{1 + {\mathrm{e}}^{ - B_i}}}} \right),\\ B_i|X_i,a,b,c,d\sim {\mathrm{GP}}\left( {0,k\left( {X_i,x} \right)} \right),\\ k\left( {x_1,x_2} \right) = {\mathrm{exp}}\left( {a - \frac{{\left( {x_1 - x_2} \right)^2}}{{b^2}}} \right) + {\mathrm{exp}}\left( {c - \frac{{\left( {x_1 - x_2} \right)^2}}{{d^2}}} \right),\end{array}$$

where GP refers to a Gaussian process, *k*(*x*_1_, *x*_2_) is the covariance function (an unnormalized version of the correlation function discussed in the main text) and *a*, *b*, *c*, and *d* are model parameters. All observations (*Y*_*i*_ and *D*_*i*_) are conditionally independent given *B*_*i*_. A latent variable model is appropriate for modeling AB since it is a property of the amplified DNA that is only indirectly observed by sequencing reads. Because a Gaussian process is used to model AB, we allow *B*_*i*_ to range over (−∞, ∞) and convert it to a value in [0, 1] using the logistic transform $1/(1 + {\mathrm{e}}^{ - B_i})$. Although we will often refer to *B*_*i*_ as allele balance, the logistic transform must be applied to arrive at the intuitive interpretation of AB as the fraction of amplified DNA derived from one allele. The form of the covariance function is an arbitrary choice. We chose to combine two radial basis functions so that one could account for very short-range effects, which tend to inflate correlation due to shared reads between loci, and the other could account for medium- to long-range effects driven by MDA amplicon size. A noteworthy property of *k*(*x*_*i*_, *x*_*j*_) is that it gives the covariance between two AB values at positions *x*_*i*_ and *x*_*j*_ using only the distance between the two sites $\left| {x_i - x_j} \right|$.

### Fitting the allele balance correlation function

The covariance function *k* contains all model parameters. Parameters are fit separately for each chromosome by maximizing the likelihood function using a grid search. The likelihood function is

3 $$\begin{array}{l}{\cal{L}}\left( {a,b,c,d{\mathrm{|}}\overrightarrow {\bf{Y}} ,\overrightarrow {\bf{D}} ,\overrightarrow {\bf{X}} } \right) = {\int}_{{\Bbb R}^n} {\underbrace {P\left( {\overrightarrow {\bf{Y}} {\mathrm{|}}\overrightarrow {\bf{B}} ,\overrightarrow {\bf{D}} } \right)}_{{\mathrm{Binomial}}\,{\mathrm{PMF}}}\underbrace {P\left( {\overrightarrow {\bf{B}} {\mathrm{|}}\overrightarrow {\bf{X}} ,a,b,c,d} \right)}_{{\mathrm{Multivariate}}\,{\mathrm{normal}}\,{\mathrm{PDF}}}} d\overrightarrow {\bf{B}} ,\\ = {\int}_{{\Bbb R}^n} {\left( {\mathop {\prod }\limits_i {\mathrm{Bin}}\left( {Y_i; D_i,\frac{1}{{1 + {\mathrm{e}}^{ - B_i}}}} \right)} \right) \cdot {\mathrm{det}}\left( {2\pi K} \right)^{ - \frac{1}{2}}{\mathrm{exp}}\left\{ { - \frac{1}{2}\overrightarrow {\bf{B}} ^T{\bf{K}}^{ - 1}\overrightarrow {\bf{B}} } \right\}d\overrightarrow {\bf{B}} ,} \\ \propto {\mathrm{det}}\left( {2\pi {\bf{K}}} \right)^{ - \frac{1}{2}} \int_{{\Bbb R}}^{n} {\exp \left\{ {\overrightarrow {\bf{B}} ^T\overrightarrow {\bf{Y}} - \mathop {\sum}\limits_{i = 1}^n {\mathop {\sum }\limits_{i = 1}^n D_i{\mathrm{log}}\left( {1 + {\mathrm{e}}^{B_i}} \right)--\frac{1}{2}\overrightarrow {\bf{B}} ^T{\bf{K}}^{ - 1}\overrightarrow {\bf{B}} } } \right\}d\overrightarrow {\bf{B}} ,} \\ \left( {\bf{K}} \right)_{i,j} = k\left( {X_i,X_j} \right).\end{array}$$

Here, *n* denotes the number of hSNPs on the chromosome being fit (which typically ranges from 10^4^ to 10^5^) and the parameters *a*, *b*, *c*, and *d* are required to calculate the covariance matrix *K*, but are suppressed for brevity. The vectors $\vec Y,\vec D,{\mathrm{and}}\,\vec X$ contain all *n* observations on the chromosome being fit. Computing this likelihood function is difficult: the integrand has no closed form solution and is also impractical to approximate numerically because it involves integrating over the very high dimensional space ${\Bbb R}^n$. We therefore employ two approximations to compute ${\cal{L}}$ in reasonable time: (1) each chromosome is divided into non-overlapping blocks of 100 hSNPs, which are treated as independent, and (2) the Laplace approximation is applied to estimate the reduced-dimension integral. The resulting approximation for a single chromosome is

4 $${\cal{L}}\left( {a,b,c,d{\mathrm{|}}\overrightarrow {\bf{Y}} ,\overrightarrow {\bf{D}} ,\overrightarrow {\bf{X}} } \right) \approx \mathop {\prod }\limits_{b = 1}^{n/100} \hat {\cal{L}}_{{\mathrm{Laplace}}}\left( {a,b,c,d{\mathrm{|}}\overrightarrow {\bf{Y}} _b,\overrightarrow {\bf{D}} _b,\overrightarrow {\bf{X}} _b} \right),$$

where the vectors $\vec {\bf{Y}}_b,\vec {\bf{D}}_b,\vec {\bf{X}}_b$ refer to observations for the *b*th block of 100 hSNPs. The basic idea behind the Laplace approximation is to replace the intractable integrand with an easy-to-integrate Gaussian function. To do this, a second order Taylor expansion around the maximizer of the unapproximated distribution is used, giving the multivariate normal posterior

5 $$\begin{array}{l}\overrightarrow {\bf{B}} _{{\mathrm{hSNP}}}|\overrightarrow {\bf{Y}} ,\overrightarrow {\bf{D}} ,\overrightarrow {\bf{X}} \sim {\mathrm{MVN}}\left( {\widehat {\bf{B}}_{{\mathrm{hSNP}}},\left( {{\bf{K}} + {\bf{W}}^{ - 1}} \right)^{ - 1}} \right),\\ \widehat {\bf{B}}_{{\mathrm{hSNP}}} = \mathop {{{\mathrm{arg}}\,{\mathrm{max}}}}\limits_{\vec B \in {\Bbb R}^n} \left\{ {\left( {\mathop {\prod }\limits_i {\mathrm{Bin}}\left( {Y_i; D_i,\frac{1}{{1 + {\mathrm{e}}^{ - B_i}}}} \right)} \right) \cdot {\mathrm{det}}\left( {2\pi {\bf{K}}} \right)^{ - \frac{1}{2}}{\mathrm{exp}}\left\{ { - \frac{1}{2}\overrightarrow {\bf{B}} ^T{\bf{K}}^{ - 1}\overrightarrow {\bf{B}} } \right\}} \right\},\\ {\bf{W}} = - \nabla \nabla _{\vec B}\log P\left( {\overrightarrow {\bf{Y}} |\widehat {\bf{B}}_{{\mathrm{hSNP}}},\overrightarrow {\bf{D}} } \right).\end{array}$$

To compute these values and the final approximate log likelihood, we use algorithm 3.1 from ref. ^20^ with the modification

6 $$\log P\left( {Y_i{\mathrm{|}}B_i,D_i} \right) = Y_iB_i - D_i\log \left( {1 + {\mathrm{e}}^{B_i}} \right).$$

The maximizer $\hat {\bf{B}}_{{\mathrm{hSNP}}}$ is approximated by Newton-Raphson iteration. Iteration continues until the *n*th iteration’s estimate of the log-likelihood function log *P*^(n)^ satisfies

7 $$\frac{{\left| {\log P^{(n)} - \log P^{(n - 1)}} \right|}}{{\left| {\log P^{(n)}} \right|}} \le \varepsilon ,$$

with $\varepsilon = \sqrt {2.2 \times 10^{ - 16}}$ or the number of iterations exceeds *n* = 50.

### Predicting allele balance at somatic SNV candidates

AB at each sSNV candidate locus is predicted following algorithm 3.2^20^, except that we are interested only in the mean and variances of the latent GP. Only hSNPs within 200 kb of each candidate sSNV are considered. Since this is an order of magnitude longer than typical MDA amplicon sizes, all relevant hSNPs should be included. Like the fitting process, prediction also requires an approximation of the true distribution due to analytical intractability. First, hSNP ABs in the window centered at the candidate sSNV are approximated by the Laplace approximation described in the previous section, which provides $\hat {\bf{B}}_{{\mathrm{hSNP}}}$ and the Hessian **W**. The posterior distribution of the AB at candidate location *X*_sSNV_ is then the normal distribution given by:

8 $$\begin{array}{l}B_{{\mathrm{sSNV}}}|X_{{\mathrm{sSNV}}},\widehat {\bf{B}}_{{\mathrm{hSNP}}},\overrightarrow {\bf{X}} \sim N\left( {\mu ,\sigma ^2} \right),\\ \mu = k\left( {X_{{\mathrm{sSNV}}},\overrightarrow {\bf{X}} } \right)^T{\bf{K}}^{ - 1}\widehat {\bf{B}}_{{\mathrm{hSNP}}},\\ \sigma ^2 = k\left( {X_{{\mathrm{sSNV}}},X_{{\mathrm{sSNV}}}} \right) - k\left( {X_{{\mathrm{sSNV}}},\overrightarrow {\bf{X}} } \right)^T\left( {{\bf{K}} + {\bf{W}}^{ - 1}} \right)^{ - 1}k\left( {X_{{\mathrm{sSNV}}},\overrightarrow {\bf{X}} } \right).\end{array}$$

### Allele balance consistency test

The allele balance consistency (ABC) test ensures that candidate sSNVs reasonably match the local AB. The AB model estimates the fraction of amplicons derived from one of the two alleles, but a somatic mutation could occur on either. We therefore choose the AB that most closely matches the VAF of the candidate sSNV using the heuristic

9 $$\mu ^ \ast \equiv \mathop {{{\mathrm{arg}}\,{\mathrm{min}}}}\limits_{x \in \left\{ {\mu , - \mu } \right\}} \left\{ {\left| {{\mathrm{VAF}} - \frac{1}{{1 + {\mathrm{e}}^{ - x}}}} \right|} \right\}.$$

The ABC null model is then

10 $$\begin{array}{l}Y|B\sim {\mathrm{Bin}}\left( {D,\frac{1}{{1 + {\mathrm{e}}^{ - B}}}} \right),\\ B|\mu ^ \ast ,\sigma ^2\sim N\left( {\mu ^ \ast ,\sigma ^2} \right).\end{array}$$

The probability of *y* reads supporting the sSNV is found by marginalizing over the posterior AB distribution

11 $$P\left( {Y = y|\mu ^ \ast ,\sigma ^2} \right) = {\int} {P\left( {Y = y|B = b} \right) \cdot P\left( {B = b|\mu ^ \ast ,\sigma ^2} \right){\mathrm{d}}b}$$

12 $$P\left( {Y = y|\mu ^ \ast ,\sigma ^2} \right) = {\int_{ - \infty }^\infty} {\left( {\begin{array}{*{20}{c}} D \\ y \end{array}} \right)\left( {\frac{1}{{1 + {\mathrm{e}}^{ - b}}}} \right)^y\left( {\frac{{{\mathrm{e}}^{ - b}}}{{1 + {\mathrm{e}}^{ - b}}}} \right)^{D - y}\frac{1}{{\sqrt {2\pi \sigma ^2} }}{\mathrm{e}}^{ - \frac{{(b - \mu ^ \ast )^2}}{{2\sigma ^2}}}{\mathrm{d}}b}$$

The integral is approximated using Gauss-Hermite quadrature with 128 nodes^21^. Let *k* be the observed number of variant-supporting reads at a locus. The ABC *p*-value *p*_ABC_ is computed by summing all events with lower probability of occurrence than *k*:

13 $$p_{{\mathrm{ABC}}} = \mathop {\sum }\limits_{y = 0}^D P\left( {Y = y|\mu ^ \ast ,\sigma ^2} \right) \cdot I\left\{ {P\left( {Y = y|\mu ^ \ast ,\sigma ^2} \right) \le P\left( {Y = k|\mu ^ \ast ,\sigma ^2} \right)} \right\},$$

where *I*(·) is the indicator function.

### Artifact statistical tests

Artifact statistical tests are designed to detect scDNA-seq errors with the greatest chance of attaining high VAF: pre-amplification errors, which often occur during cell lysis, and first-round amplification errors. The artifact tests follow the ABC test except that the null model includes the possibility of the error occurring on either allele, meaning there is no need to compute μ*. Let allele 1 be the allele directly modeled by *B* and allele 2 be the other allele. Then the null artifact model is the mixture distribution given by

14 $$\begin{array}{l}Y|B,{\mathrm{allele1}}\sim {\mathrm{Bin}}\left( {D,\frac{1}{f}\frac{1}{{1 + {\mathrm{e}}^{ - B}}}} \right),\quad Y|B,{\mathrm{allele}}\,{\mathrm{2}}\sim {\mathrm{Bin}}\left( {D,\frac{1}{f}\left( {1 - \frac{1}{{1 + {\mathrm{e}}^{ - B}}}} \right)} \right)\\ P(Y|B) = \frac{1}{2}P\left( {Y|D,{\mathrm{allele}}\,{\mathrm{1}}} \right) + \frac{1}{2}P\left( {Y|D,{\mathrm{allele}}\,{\mathrm{2}}} \right).\end{array}$$

For pre-amplification artifacts *f* = 2, meaning that a pre-amplification artifact is expected to occur on half of amplicons derived from the artifact-harboring allele. This corresponds to two assumptions: (1) pre-amplification artifacts are single-stranded and (2) the two strands are equally amplified. Amplification artifacts are modeled with *f* = 4, reflecting the following ideal amplification scenario. DNA from one allele is initially double-stranded. Suppose both strands of the allele are fully replicated exactly once before any other replication occurs, producing four strands of DNA. Polymerase incorporation errors occurring during this process will therefore be present on one-fourth of strands from this allele. Assume amplification of the four strands continues without additional errors, eventually producing approximately equal numbers of amplicons from each strand. A misincorporation error in the first round will therefore be supported by one-quarter of molecules in the amplicon pool for this allele.

### Estimating artifact prevalence

Under certain assumptions, it is possible to bound the number of true sSNVs in a set of candidate sSNVs using a large enough set of high confidence hSNPs. The necessary assumptions are that true sSNVs are present on 50% of DNA molecules prior to amplification (e.g., as would occur for a fully diploid genome) and that true sSNVs are not too concentrated in specific genomic regions, so that the sSNV VAF distribution resembles hSNP VAFs. Under these assumptions, the fraction of somatic SNVs at a specific VAF should not be too different from the fraction of hSNPs at that same VAF. Using hSNPs as a guide, the expected number of true sSNVs at each VAF can be computed for any *N*_T_ > 0. When *N*_T_ is too large, the predicted number of true sSNVs for some VAFs will exceed the number of candidate sSNVs at that VAF, making it evident that the chosen *N*_T_ is likely inconsistent with the data.

We use a multinomial simulation to evaluate the consistency of several possible values of *N*_T_ with the observed hSNPs and candidate sSNVs. Only successfully phased hSNPs are used for these simulations. hSNPs *H*_*v*_ and sSNVs *S*_*v*_ falling into 20 equally sized VAF bins are counted such that:

15 $$\begin{array}{*{20}{l}} {H_1 = \# \left\{ {{\mathrm{hSNPs}}\hskip -3pt:0.00 \le {\mathrm{VAF}} \, < \, 0.05} \right\},} \hfill & {} \hfill & {S_1 = \# \left\{ {{\mathrm{sSNVs}}\hskip -3pt:0.00 \le {\mathrm{VAF}} \, < \, 0.05} \right\},} \hfill \\ {H_2 = \# \left\{ {{\mathrm{hSNPs}}\hskip -3pt:0.05 \le {\mathrm{VAF}} \, < \, 0.10} \right\},} \hfill & {} \hfill & {S_2 = \# \left\{ {{\mathrm{sSNVs}}\hskip -3pt:0.05 \le {\mathrm{VAF}} \, < \, 0.10} \right\},} \hfill \\ {} \hfill & \vdots \hfill & {} \hfill \\ {H_{20} = \# \left\{ {{\mathrm{hSNPs}}\hskip -3pt:0.95 \le {\mathrm{VAF}} \le 1.00} \right\},} \hfill & {} \hfill & {S_{20} = \# \left\{ {{\mathrm{sSNVs}}\hskip -3pt:0.95 \le {\mathrm{VAF}} \le 1.00} \right\}.} \hfill \end{array}$$

We assess the consistency of any value of *N*_T_ > 0 with the data as follows. First, we simulate the numbers of sSNVs in each VAF bin 1000 times by drawing from a multinomial distribution with parameters matching the binned hSNPs

16 $$\left\{ {S^{\left( i \right)}\sim {\mathrm{Multinom}}\left( {N_{\mathrm{T}},\frac{{H_1}}{H}, \cdots ,\frac{{H_{20}}}{H}} \right)} \right\}_{i = 1}^{1000},\quad H = \mathop {\sum }\limits_{v = 1}^{20} H_v.$$

The fraction $F_{N_{\mathrm{T}}}$ of simulations consistent with the observed sSNV candidate counts evaluates the fit of *N*_T_ to the data.

17 $$F_{N_{\mathrm{T}}} = \frac{1}{{1000}}\mathop {\sum }\limits_{i = 1}^{1000} I\left( {S_1^{(i)} \le S_1 \wedge \cdots \wedge S_{20}^{(i)} \le S_{20}} \right).$$

This process is repeated for several values of *N*_T_ ranging from 1 to the total number of sSNV candidates. The upper bound on the somatic mutation burden is the largest *N*_T_ such that $F_{N_{\mathrm{T}}} \ge 0.005$. We choose such a lenient criterion to account for the fact that inconsistency occurs even if only a single VAF bin out of 20 exceeds the candidate sSNV count at that VAF.

Given the estimate of *N*_T_, the expected numbers of true sSNVs and artifacts in VAF bin *i* are

18 $$N_{{\mathrm{T}},i} = N_{\mathrm{T}}\frac{{H_i}}{H},\quad N_{{\mathrm{A}},i} = \max \left\{ {N_{\mathrm{T}}\left( {1 - \frac{{H_i}}{H}} \right),0.1} \right\}.$$

Since these values represent expectations, they may be <1. The arbitrary minimum value of 0.1 for the artifact burden is to avoid FDR estimates of 0%.

The preceding procedure is applied separately to sSNVs and hSNPs with the same sequencing depth; all sSNVs and hSNPs with depth greater than the 90th percentile of hSNPs are treated together. This produces *N*_T*,i,D*_ and *N*_A*,i,D*_, which estimate the number of true mutations and artifacts for each VAF and depth.

### Determining *p*-value cutoffs

The ABC test uses a fixed cutoff of 0.05. *p*-value cutoffs for both artifact tests are tuned at every sSNV candidate to obey a user-supplied false discovery rate *θ*. This is possible because the alternative hypothesis of an artifact test—that the site is a true mutation—provides a specific model with which statistical power (1 − *β*) can be computed for any *p*-value cutoff *α*. Highest density regions^22^ are used to account for the multi-modal nature of the artifact models and the integer read counts. Given the artifact prevalence estimates from the previous section for the VAF and depth of the candidate sSNV, the expected FDR for a *p*-value cutoff *α* can be computed using the relationship provided in the main text. The largest *α* satisfying the requested FDR *θ* at each sSNV is used. This procedure does not formally control the FDR, as this would require formal models of *N*_T_ and *N*_A_ rather than the heuristics used here.

### Multi-sample calling

SCAN-SNV’s joint calling model is based on the idea that true mutations should match the estimated AB in every sample in which they are observed. We define a joint statistic *J* as the product of ABC *p*-values for all samples with any mutation supporting reads. Each ABC *p*-value is subject to a penalty factor of 1/10 if the standard deviation of the GP at the locus exceeds 1. *J*-statistics are also computed for 4000 randomly selected hSNPs which are then grouped based on the number of single cells supporting the hSNP. Ninetieth percentiles are computed for each group of hSNP *J*-statistics and used as thresholds for sSNVs supported by the same number of samples. Unlike single-sample calls, joint calls are not filtered by the artifact statistical tests or excess indel and read clipping filters.

### Excess indel and read clipping filters

Artefactual sSNVs may result from nearby misalignments or areas of poor alignment. These may be single-cell specific if, e.g., an MDA chimera^23^ event occurred. One sign that a locus may be compromised in a single cell is a large number of sequencing reads with indels or soft- or hard clipping which is not also seen in the matched bulk. BWA-MEM reports these events in the CIGAR string as D, I, S, and H operations. We therefore sought to determine how many indel (I, D) or clipping (H, S) operations should be tolerated at somatic SNV candidates by comparing to the rate of the same CIGAR operations in bulk at the same locus. We therefore compute the fraction of reads containing indel and clip operations for both bulk and single cell at each candidate sSNV and a set of 4000 randomly chosen hSNPs. The hSNPs are used to build 2-dimensional empirical distributions (*F*_Single cell,OP_, *F*_Bulk,OP_), where *F*_T,OP_ is the fraction of CIGAR operations of type OP (either indel or clip) spanning a single hSNP in the specified sample type *T*. Similar quantities are computed for each candidate somatic SNV. Candidates are filtered if they exceed the 90th percentile of either (i.e., indel or clip) hSNP empirical distribution.

### Running SCAN-SNV

SCAN-SNV is implemented using Snakemake^24^ and distributed as a Conda^25^ package. SCAN-SNV and all dependencies were installed using Conda into a blank environment and the scansnv script was run with default parameters. External databases used were the human reference genome b37d5 (--ref), dbSNP 147 common variants (--dbsnp), and the 1000 Genomes Project phase 3 with X chromosome dated October 2014 provided by SHAPEIT2 (--shapeit-panel). SCAN-SNV was run on a SLURM cluster using the --drmaa flag. Candidate sSNVs that pass the ABC test, pre-amplification and amplification artifact tests using a target FDR of 10%, and excess indel and clipping tests are reported.

### Running other somatic SNV callers

SCcaller version 1.1 was run as previously reported^10^. BAMs were converted to pileups using samtools version 1.3.1 with the option -C50 and hSNPs were defined using dbSNP version 146. Single cell somatic SNVs were called by applying SCcaller’s -a varcall, -a cutoff and reasoning v1.0 script in sequence with default parameters. As recommended on the Github README, passing somatic mutations were required to have VAF > 1/8, filter status = PASS, bulk status = refgenotype and must not have been observed in dbSNP. The stringent artifact threshold corresponding to *α* = 0.01 was used for assessment.

Monovar commit 7b47571 was downloaded and the somatic calling strategy reported previously^9^ was mimicked as closely as possible; no script is provided for identifying somatic mutations. Single cell BAMs were input to samtools version 1.9 with options -BQ0 -d10000 -q 40, which was piped into the monovar.py script with options -p 0.002 -a 0.2 -t 0.05 -m 2 as recommended by the authors. To determine whether SNVs were somatic or germline, samtools was run with the same options on matched bulk data. Somatic SNVs were determined by the following filters: Monovar’s genotype string must not match ./. or 0/0; minimum sequencing depth of 10 with at least 3 reads supporting the mutation; at least 6 reads in bulk with no more than 1 mutation supporting read; and single-cell VAF ≥ 10% for sSNVs with >100 depth or VAF ≥ 15% for sSNVs with depth between 20 and 100. Finally, sSNVs were filtered if any other call occurred within 10 bp. For joint calling, the VCF column FILTER=PASS was also required.

MuTect calls were produced by MuTect version 1.1.7. Although MuTect 2 is now preferred, it relies on an external panel of normals for proper filtration. We chose to use MuTect 1 because it is unclear to what extent MuTect 2 performance would depend upon this panel and because no reference panel of normals is publicly available. MuTect was run with default parameters except -dt None and COSMIC v72^26^ and dbSNP 147 (common variants) were passed to parameters --cosmic and --dbsnp, respectively. All sSNVs with FILTER=PASS were retained.

GATK HaplotypeCaller was run jointly on all SD samples and mixed bulk with the extra parameters -mmq 60 -rf BadCigar --dontUseSoftClippedBases followed by VQSR with -mode SNP and GATK best practices parameters for the HapMap, Omni, 1000 Genomes and dbSNP databases. Somatic SNVs were filtered from the raw calls by requiring 0 mutation supporting reads, depth ≥ 10 and genotype string 0/0 in bulk, any single-cell genotype string other than ./. and 0/0, absence from dbSNP 147 common and a VQSR tranche status of at least 90% or 99% for sensitivity targets 90% and 99%, respectively.

### Synthetic diploid read mixing

Aligned and processed BAMs for individuals 5087 and 5532 were downloaded from dbGaP study phs001485.v1.p1. Reads aligned to chromosome X were extracted from each BAM and downsampled to an average of 15× using samtools view’s -s option. Spike-in mutations were then added to each downsampled BAM as described below. Finally, a downsampled, spike-in carrying BAM from each individual was mixed with samtools merge to create a single BAM with ~30× mean depth (Supplementary Table 2).

### Synthetic diploid spike-ins

First, a genomic blacklist was created to ensure that spike-in mutations would not intersect with endogenous variants or artifacts, pseudoautosomal regions (PARs), assembly gaps or common variant sites as reported by dbSNP 147. To identify potential endogenous variants and artifacts, GATK HaplotypeCaller was run jointly on all full-depth BAMs with default parameters. A 5 bp window centered at each position with non-reference reads output by GATK was added to the blacklist. Similarly, 5 bp windows centered at the position of every record in dbSNP 147 common were also added. Finally, the blacklist was completed by adding 26 assembly gaps annotated by UCSC (hgdownload.cse.ucsc.edu/goldenPath/hg19/database/gap.txt.gz) and the hg19 coordinates for PAR1 (60,001–2,699,520) and PAR2 (154,931,044–155,260,560). In total, 3750 spike-in mutations (corresponding to 15 branches with 250 mutations each) were created by choosing random positions from the non-blacklisted genome and a random non-reference base. Two hundred and fifty mutations were assigned to each branch of the phylogeny and spiked into one of the two downsampled donor BAMs for all descendent SDs using BAMSurgeon (Supplementary Table 2). Spike-ins were added at 100% VAF. A spike-in was considered successful if at least 1 alternate read survived BAMSurgeon’s mutation and realignment process. The number of successful spike-ins ranged from 180 to 446, depending primarily on the breadth of coverage of each single cell. BAMSurgeon was run with the following parameters: --force --mindepth 0 --maxdepth 10000 --minmutreads 0 --ignoresnps --aligner mem. BAMSurgeon was modified by adding the parameters -I 400,90 to the BWA-MEM command because BWA often cannot infer insert size characteristics in the small windows around spike in mutations. Without this addition, the PROPER_PAIR BAM flag is often not set, causing some mutation-carrying reads to be ignored by samtools. Actual insert size mean and standard deviations ranged from 388 to 428 and 81 to 93, respectively, across all donor chromosome X BAMs.

### Synthetic diploid assessment

Sensitivity was calculated as the fraction of successful spike-ins recovered. The number of FPs per SD was the number of sSNV calls that were not known spike-ins or endogenous sSNVs. Endogenous sSNVs were determined by examining the full-depth single cell and bulk BAMs with samtools mpileup with -q 60 at every site called by any genotyper. A site was considered a putative endogenous sSNV if either: (1) the mean VAF of the sSNV across all samples with at least 2 alternate reads was ≥80% and bulk contained at least 2 reads at the locus with none supporting the sSNV; or (2) the mean VAF of the sSNV across all samples with at least 2 alternate reads was ≥90%, at least two single cells supported the sSNV and the bulk contained no reads at the locus.

### Kindred cell system

Aligned and processed BAMs for the kindred system were downloaded from the NCBI Sequence Read Archive (SRA) using accession number SRP067062. All 13 BAMs were analyzed jointly by GATK HaplotypeCaller (with -rf BadCigar --dontUseSoftClippedBases) and bcftools call (with -mvV indels -P 0.5) using minimum mapping qualities (MQs) of both 1 and 60. The -P 0.5 option was chosen to greatly increase samtools’ sensitivity. All sites output by samtools and GATK with MQ ≥ 60 non-reference reads in any sample were classified using the following criteria. First, sites were classified as supported in bulk if mutation supporting reads were observed in sample SRR2976567 in either of the MQ = 60 or MQ = 1 runs. Sites with no mutation supporting reads across all ten non-kindred samples and at least 50 total reads across all samples were considered kindred exclusive and further separated into those supported by 1, 2, or 3 of the kindred samples. Kindred exclusive sites supported by all three samples are designated triple exclusive (TRE). An additional class consisting of sSNVs supported by all three kindred samples and at most 1 supporting read in a non-kindred sample was also created to account for sequencing errors since base quality scores were not considered. All remaining sites were classified as FPs. Site classifications were further refined by a mock kindred group analysis (described below). Finally, samtools and GATK classifications were integrated according to Supplementary Table 2 to create the TRE, likely TP, likely FP and unknown designations in Fig. 6. In total, 569 TRE sites were used to compute sensitivity.

### Mock kindred group analysis

Clonal sSNVs or artifacts may be exclusive to the kindred group due to chance alone (e.g., due to chance dropout in the remaining samples or differences in read lengths). We therefore applied the same classification procedure described above to mock kindred groups consisting of three randomly chosen samples. All possible mock groups were created except the group of three clonally expanded samples and groups containing heat lysed single cells. For each mock kindred exclusive mutation supported by two or three samples, we counted the total number of mutation supporting reads across the mock kindred group. Cutoffs corresponding to the 99th (samtools) and 75th (GATK) percentiles of total mutation supporting reads were separately computed for mock sites supported by two or three of the mock kindred samples. Finally, kindred exclusive sites from the true kindred group were classified as filtered if the total number of mutation supporting reads across the kindred group did not exceed the appropriate cutoff. For GATK, cutoffs were 5 and 7 for kindred exclusive sites with two or three supporting kindred samples, respectively. Samtools cutoffs were 5 and 9. See Supplementary Fig. 1 for a detailed mock analysis of TREs.

### Increased DNA content simulation

BAMs for two single-cell samples with imbalanced hSNP VAF distributions (IL-12 and IL-3) were downsampled by 50% and combined into a single BAM using samtools. The two other imbalanced samples, HL-1 and HL-2, were not used because a different, heat-based cell lysis protocol was used that may have affected the shapes of their VAF distributions. Downsampling was repeated 10 times to account for potential variance due to read sampling. AB models were fit by SCAN-SNV on chromosome 1 for each mixed BAM. The 10 replicates are summarized by a single, representative curve in Fig. 7 since essentially no variation between the trials was observed.

### Cancer analysis

Raw FASTQ files for four ERBC tumor cells (BC1-4), tumor bulk (BCT) and matched normal bulk (BCN) were downloaded from the NCBI SRA, accession SRP013572. Reads were aligned with BWA-MEM to GRCh37 with decoy and postprocessed using Picard MarkDuplicates, GATK indel realignment and GATK base quality score recalibration. Putative sSNVs from all callers were gathered and samtools mpileup -q 60 was used to determine read support for each mutation across all six samples. sSNVs were then classified according to Supplementary Table 3. Haploid, diploid, and triploid chromosomes were identified by previously reported copy number profiles obtained by sequencing the bulk tumor population and 50 single cells^1^.

### Reporting summary

Further information on research design is available in the Nature Research Reporting Summary linked to this article.

## Supplementary information

Supplementary Information

Reporting Summary

## Data Availability

Source single-cell sequencing data for synthetic diploid X chromosomes were downloaded from dbGaP study phs001485.v1.p1. Single-cell sequencing data for the kindred cell system were downloaded from NCBI SRA project SRP067062. Sequencing data for single tumor cells were downloaded from NCBI SRA project SRP013572. All other relevant data are available upon request.

## Code availability

Source code for SCAN-SNV is available for download at https://github.com/parklab/scan-snv. SCAN-SNV version 0.9 and r-scansnv version 0.1 were used to produce all results in this study.
