# Supplementary material for: Identification of somatic mutations in single cell DNA-seq using a spatial model of allelic imbalance
Source: Nat Commun. 2019 Aug 29;10:3908. doi: 10.1038/s41467-019-11857-8 (PMC6715686; doi:10.1038/s41467-019-11857-8)
Supplement: Supplementary file 1 — Supplementary Information [file 41467_2019_11857_MOESM1_ESM.pdf]

Supplementary Information

Identification of somatic mutations in single cell DNA-seq using a spatial model of allelic imbalance

Luquette et al.

Correspondence to Peter J. Park (peter_park@hms.harvard.edu)

Supplementary Figure 1

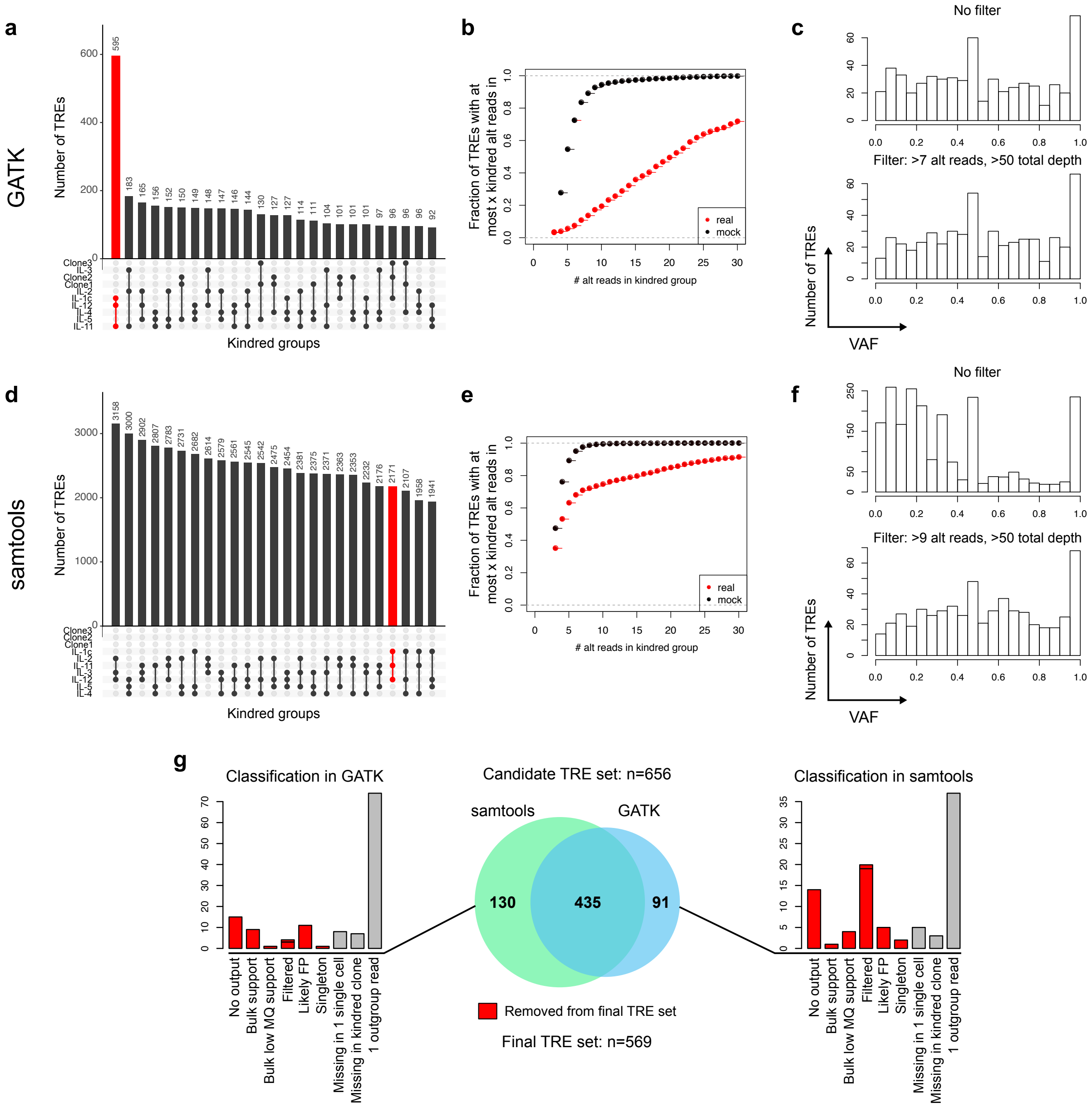

Supplementary Figure 1. Mock kindred analysis of TRE sites in kindred cells.
To remove spurious TREs, a mock kindred analysis in which random sets of 3 samples were treated as kindred groups was performed for GATK (a-c) and samtools (d-f). A similar analysis was performed for sites supported by only two kindred samples.
(a) Number of TREs detected for each kindred group (red, true kindred group; black, mock kindred groups). Top 25 mock groups by number of TREs are shown.

(b) For each TRE detected in the real or mock kindred groups, we computed the number of mutation supporting reads in the respective kindred group. Cumulative distribution functions for number of mutation reads are shown for all mock TREs (black) and true TREs (red).
(c) VAF distribution of the TRE set in kindred cell IL-12 before (top) and after (bottom) filtration. Number of alternate reads for filtering refers to the total number of alternate reads shared by the kindred group.
(d-f) Same as (a-c) applied to samtools.
(g) Merging GATK and samtools TREs to create the final set. TREs specific to one caller were retained for the final set if the other caller found no more than 1 mutation read out of the kindred group and support in at least 2 kindred samples.

Supplementary Figure 2

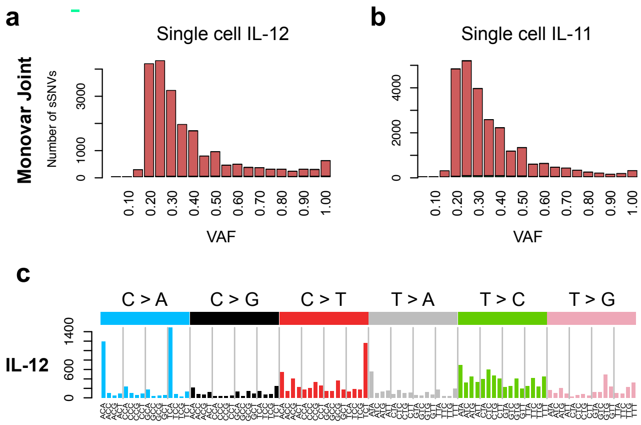

Supplementary Figure 2. Joint Monovar performance on kindred cells.
VAF breakdowns of joint Monovar calls for kindred cells IL-12 **(a)** and IL-11 **(b)**, similar to main Figure 5d,g. **(c)** Trinucleotide mutation signature of joint Monovar sNVs in IL-12.

Supplementary Figure 3

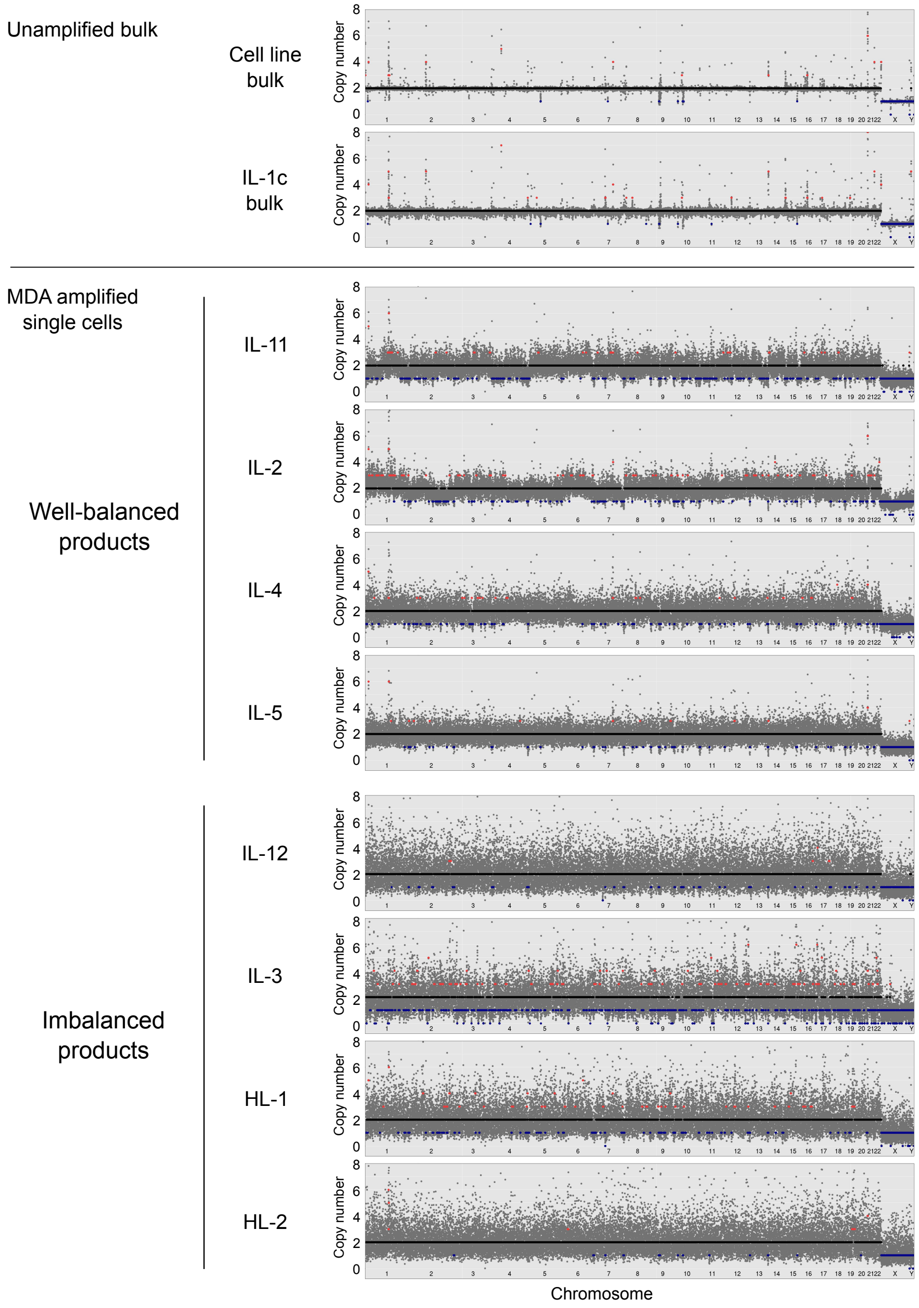

Supplementary Figure 3. Copy number profiles for kindred analysis samples.
Copy number profiles were computed by Ginkgo for the cell line bulk and kindred clone bulk (IL-1c) as well as all MDA-amplified single cells. Single cells are grouped according to the well-balanced and imbalanced designations shown in Fig. 6a. Well-balanced products tend to have less variability in copy number than imbalanced products.

Supplementary Figure 4

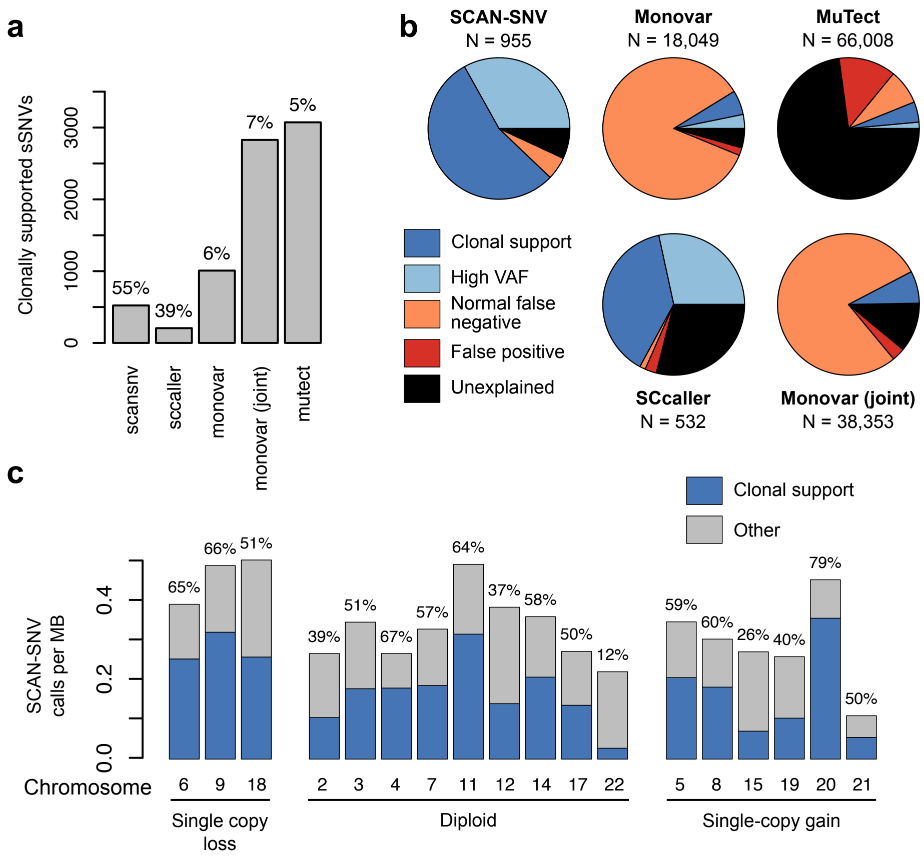

Supplementary Figure 4. Analysis of 4 single ERBC tumor cells.
(a) Total number of sSNVs that were supported by bulk sequencing of the same tumor cell population or multiple single cells. The percent of the total callset made up by these clonally supported calls is displayed above each bar.
(b) Classification of all calls made by each method according to Supp. Table 3.
(c) Total SCAN-SNV sSNV calls per chromosome, grouped by whether the chromosome is clonally haploid, diploid or triploid in the tumor cell population. Percent above each bar: fraction of clonally supported calls.

Supplementary Table 1

SD ID	Donor single cell	Spike-in mutations by clonality (>0 reads) 1	2	4	8	Total spike-ins
1	5087-1cp1C6	96	116	107	105	424
1	5532-1cp1E11	107	104	107	105	423
2	5087-1cp1C9	101	110	117	118	446
2	5532-1cp1E9	109	89	97	88	383
3	5087-1cp1D6	99	95	92	100	386
3	5532-1cp1H11	107	103	101	110	421
4	5087-1cp1H10b	98	107	110	105	420
4	5532-1cp1H4	113	101	91	99	404
5	5087-1cp2A2	104	99	99	103	405
5	5532-1cp1H9	103	112	113	112	440
6	5087-Lp1C5	88	90	81	99	358
6	5532-Lp1C5	86	97	92	91	366
7	5087-Rp1G4	85	73	96	89	343
7	5532-Lp2C5	68	81	79	78	306
8	5087-Rp3C5	48	51	44	37	180
8	5532-Rp1C11	60	52	67	67	246

Supplementary Table 1. Synthetic diploid samples and spike-ins. Single cells used to construct synthetic diploid (SD) X chromosomes 1-8. Each SD was assigned 1,000 spike-ins, but some failed due to, e.g., lack of reads at the locus. Number of successful sSNVs spiked into downsampled BAMs before mixing, grouped by the number of SDs sharing the sSNV, are shown. SD ID, synthetic diploid identifier.

Supplementary Table 2

		samtools									
						Filtered					
		No call	Bulk	FP	1	2	4	TRE	2	4	TRE
GATK	No call	0	216937	525839	558240	35666	2259	1509	255	47	15
	Bulk	52297	3481245	13783	2025	173	52	25	7	2	10
	FP	10718	3873	26281	6821	88	77	12	56	23	11
	1	3286	282	9247	11584	508	26	15	55	0	1
Filtered	2	197	30	110	124	190	3	10	10	0	1
	4	29	10	23	1	3	14	4	0	0	0
	TRE	36	4	10	2	3	0	9	2	0	3
	2	31	0	16	54	20	4	0	215	1	15
	4	17	3	7	1	4	3	3	2	45	74
	TRE	14	5	5	2	0	1	19	8	37	435

Supplementary Table 2. Integration of GATK and samtools assessments for kindred cells. All sites output by either samtools or GATK when run jointly on the 13 samples from Dong et al. Filtered: flagged as filtered by the mock kindred group experiment. Filtered sites can be recovered if they are high quality in the other caller. Background colors: red, likely FP; light blue, likely TP; dark blue, TRE; white, unknown. No call, no output available for the specified tool; Bulk, mutation supporting reads observed in normal bulk; FP, not assigned to any other class; 1, supported by a single sample (singleton); 2, supported by 2 kindred samples; 4, supported by all 3 kindred cells and at most by one read in another sample; TRE, supported by all 3 kindred samples and no other samples.

Supplementary Table 3

Mutation class	Reason
False positive	No mutation supporting reads with MQ >= 60 were reported by samtools.
Likely FN in normal	Either the: (1) reads supporting the mutation were found in matched normal; (2) the mutation is annotated as a **common** polymorphism in dbSNP 147; or (3) the normal bulk sample had depth < 5.
Clonal support	The mutation is also supported by at least 2 reads in either the bulk tumor data or at least one other single cell.
High VAF	Not belonging to any of the above classes, depth > 5 and VAF >= 50%.
Unexplained	Not belonging to any of the above classes.

Supplementary Table 3. Classification scheme for sSNVs in ERBC single cells. Clonal support and high VAF are judged as relatively high quality variants.
